# A rare pure embryonal rhabdomyosarcoma of the urinary bladder in an adult successfully managed with neoadjuvant chemotherapy and surgery: a case report

**DOI:** 10.1186/s13256-018-1870-1

**Published:** 2018-11-04

**Authors:** Mustapha Ahsaini, Khalid Ouattar, Hamid Azelmad, Soufiane Mellas, Jallal Eddine Ammari, Mohammed Fadl Tazi, Mohammed Jamal Fassi, Moulay Hassan Farih, Simohammed Sekal, Taoufik Harmouch

**Affiliations:** 1 Department of Urology, University Hospital Centre Hassan II, 30000 Fez, Morocco; 2 Department of Pathology, University Hospital Centre Hassan II, Fez, Morocco

**Keywords:** Rhabdomyosarcoma, Bladder, Hematuria, Adult, Chemotherapy

## Abstract

**Background:**

Rhabdomyosarcoma of the urinary bladder in adults is an extremely rare malignant neoplasm that develops from the bladder wall.

**Case presentation:**

We report our experience of a rare case of rhabdomyosarcoma of the bladder in a 45-year-old Moroccan man who was successfully managed with neoadjuvant chemotherapy and surgical excision of the mass; he was disease free at 24-month follow up.

**Conclusions:**

To the best of our knowledge, this is the first reported case of a rare embryonal rhabdomyosarcoma of the bladder that was managed with neoadjuvant chemotherapy and surgery. This is why further studies using a large number of patients with a greater longitudinal follow up will be required.

## Background

Rhabdomyosarcoma (RMS) is the most common soft tissue sarcoma found in children [1]. Approximately 15 to 20% of all cases of RMS are of genitourinary origin [2]. However, RMS of the bladder rarely occurs in adults [3]. There are few adult cases reported in the medical literature.

We report our experience of a rare case of RMS of the bladder in a 45-year-old man who was successfully managed with neoadjuvant chemotherapy and surgery; he was disease free at 24-month follow up.

## Case presentation

A 45-year-old Moroccan man of low socio-economic status, a farmer by profession, with no particular personal or family medical history and without any medications prior to diagnosis, presented to our emergency department with gross hematuria as the main symptom associated with urinary frequency. He had a history of tobacco smoking (24 pack-years) and did not consume alcohol.

His vital signs were: body temperature 37.5 °C, blood pressure 120/70 mmHg, and pulse 88 beats per minute. His physical examination revealed minimal lower abdominal pain without any mass, with a strictly normal neurological examination.

**Fig. 1:**
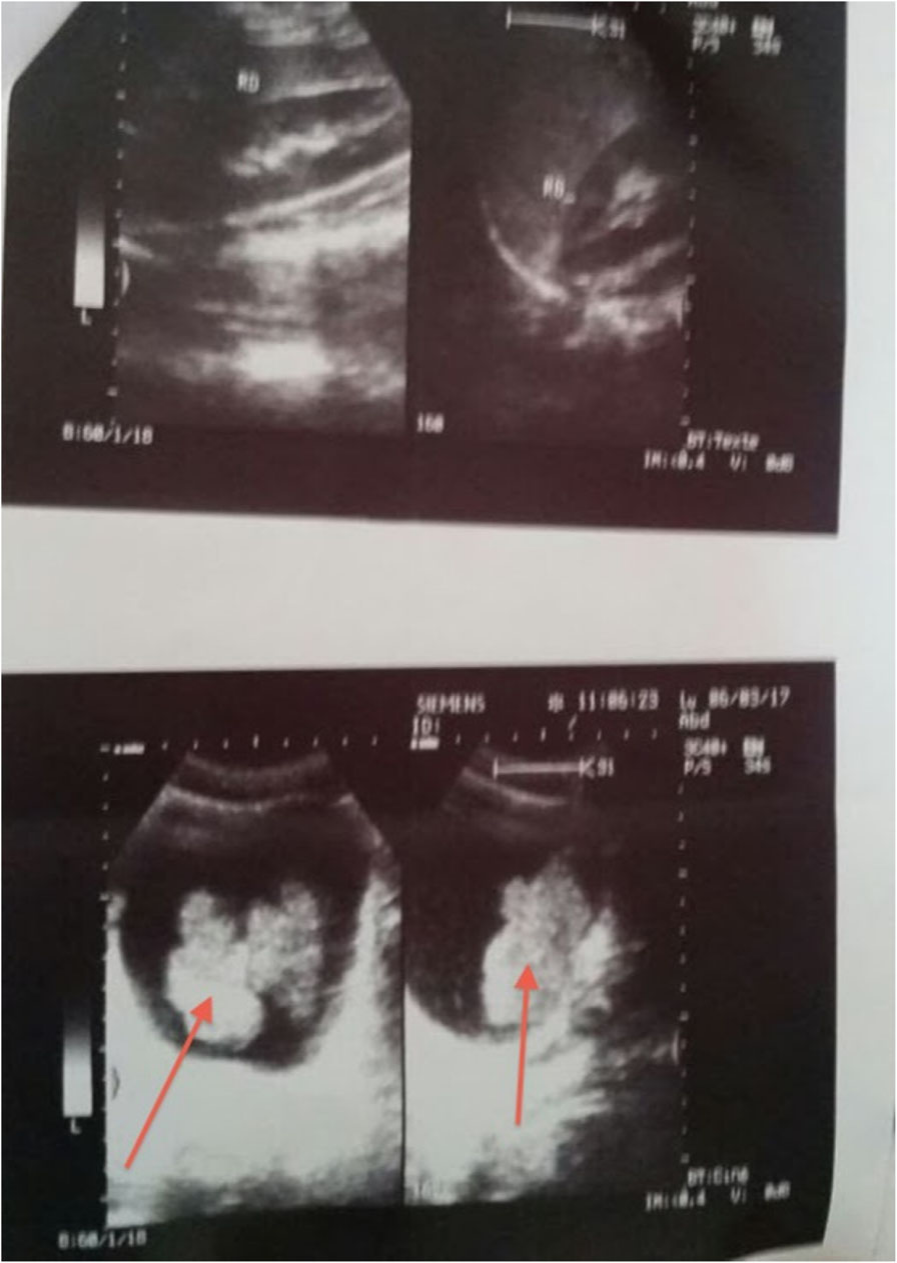
Abdomen sonography revealed a huge bladder tumor mass (70 × 60 mm, *red arrow*) at posterior wall without hydronephrosis

Laboratory data only revealed an acute anemia (hemoglobin, 8.5 g/dl) requiring four units of packed red blood cells transfusion. No other anomaly in the laboratory data was found. A urine culture was negative. An abdominal ultrasound revealed a huge mass (70 × 60 mm) on the posterior wall of the urinary bladder with no hydronephrosis (Fig. 1). Cystoscopy revealed a large endoluminal mass arising from the retrotrigonal region.

**Fig. 2:**
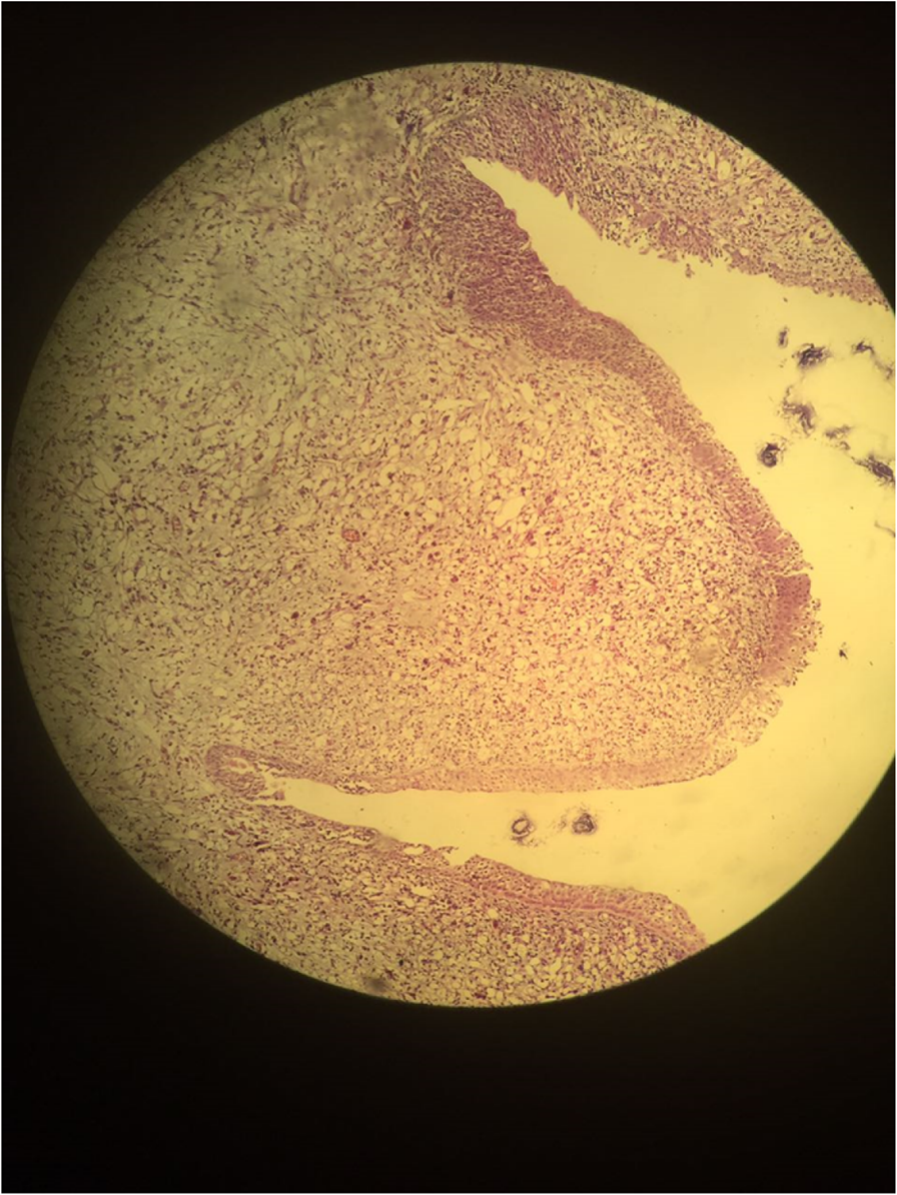
Tumor proliferation in the bladder mucosa with “cambial layer” appearance (hematein-eosin staining × 40 magnification)

**Fig. 3:**
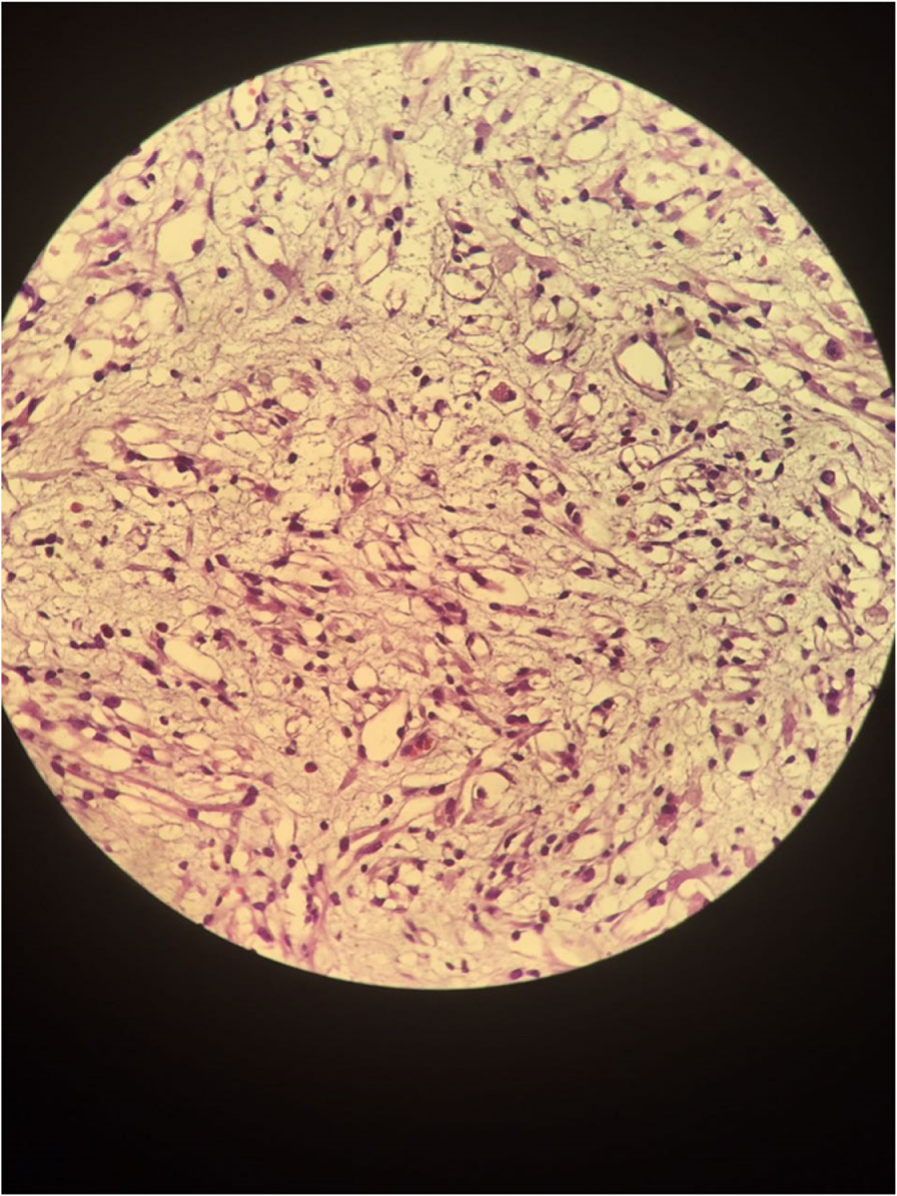
Small and round rhabdomyoblasts with reduced cytoplasm. The tumor stroma is myxoid and richly vascularized. Hematein-eosin staining, × 200 magnification

**Fig. 4:**
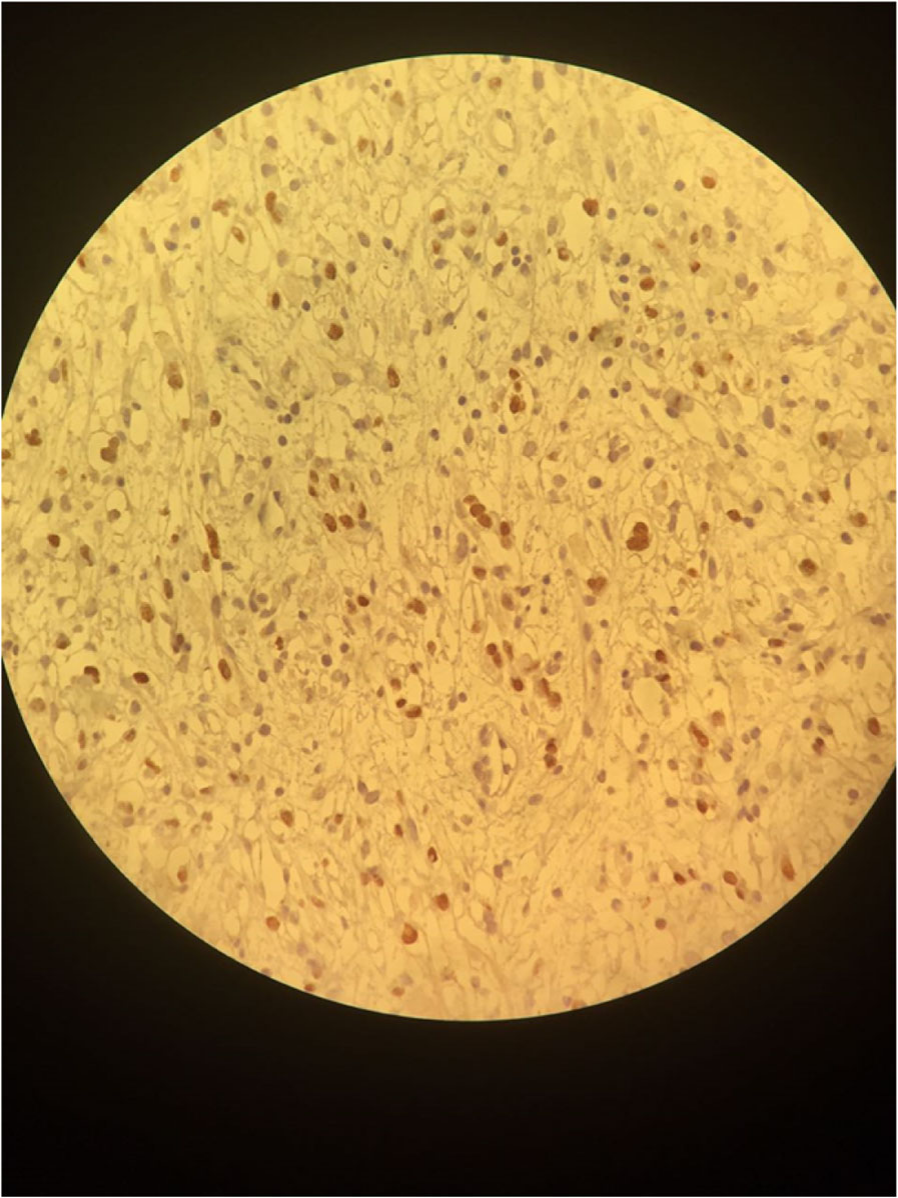
Immunohistochemistry by anti-myogenin antibody: Moderate nuclear labeling of some tumor cells

**Fig. 5:**
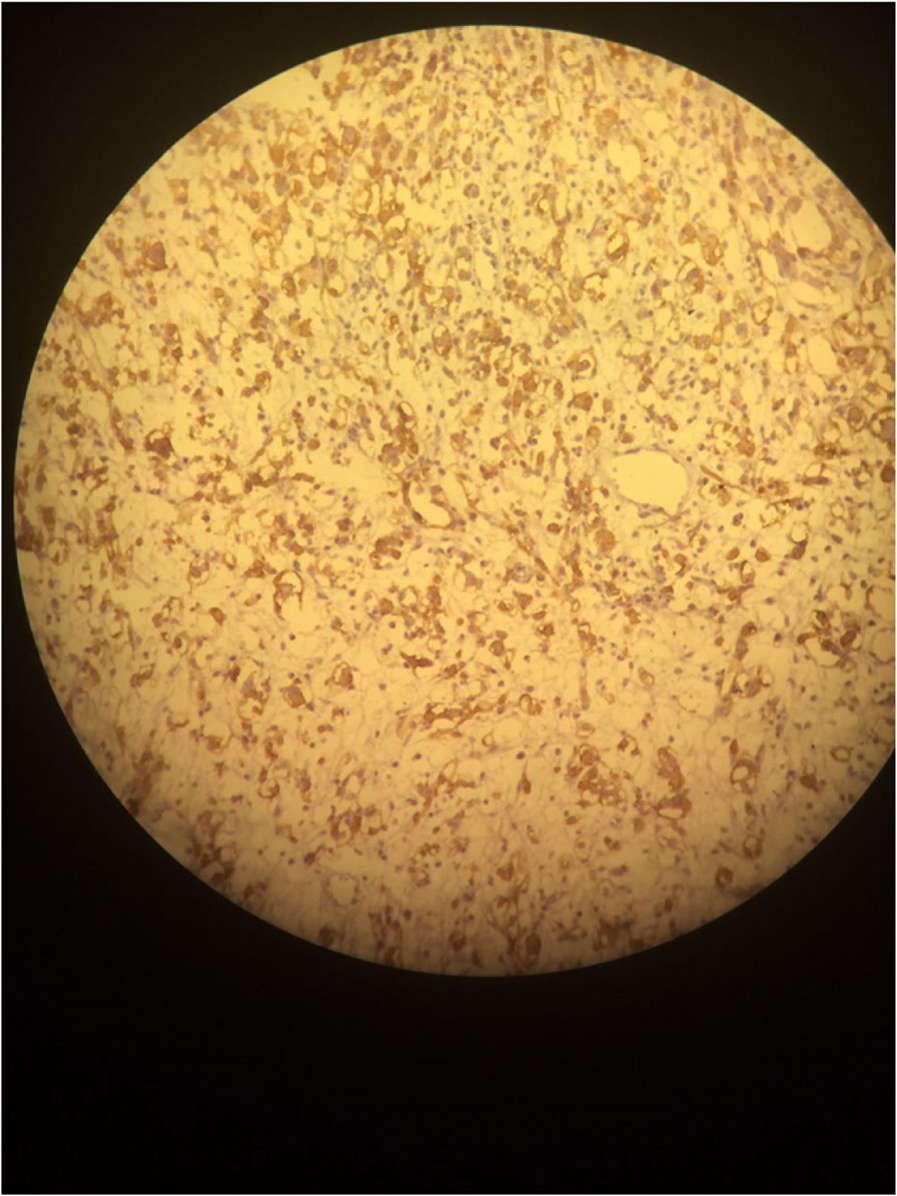
Immunohistochemistry by anti-desmin antibody: Cytoplasmic staining of tumor cells

**Fig. 6:**
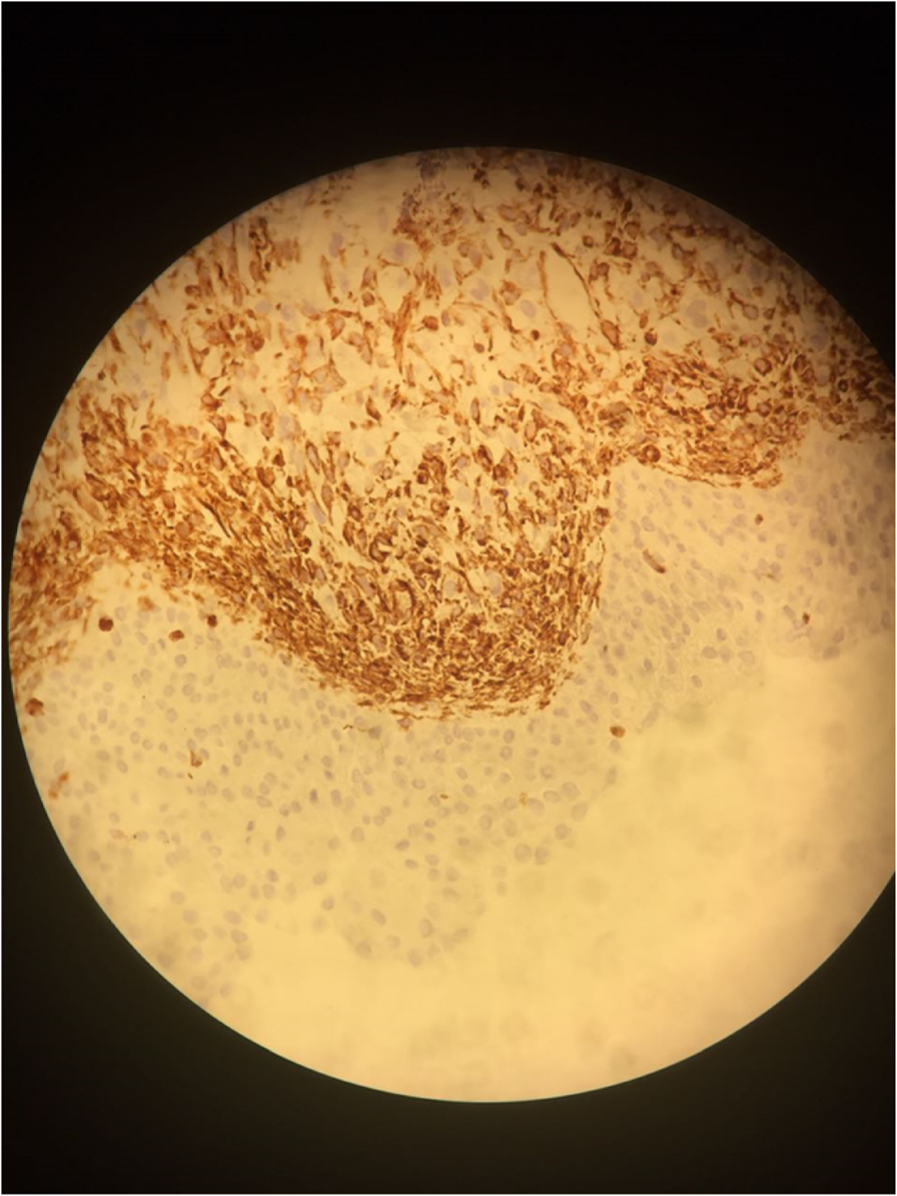
Immunohistochemistry by anti-vimentin antibody: Cytoplasmic labeling of tumor cells in the “cambial layer” and some chorion cells

A transurethral endoscopic resection of his bladder for hemostatic and biopsy purposes was performed under general anesthesia. His postoperative course was uneventful. Microscopic examination of the resected specimen showed small round cells and occasional “tennis-racket” shaped cells with acidophilic cytoplasm, the nuclei were hyperchromatic, the matrices were myxoid and richly vascularized, dense cellularity was present near the surface of the epithelium of the bladder (Figs. 2 and 3). Immunohistochemical studies showed that these cells expressed myogenin, desmin, and vimentin (Figs. 4, 5, and 6). The final diagnosis was embryonal botryoid RMS.

A thoracoabdominal computed tomography (CT) scan revealed no distant metastasis.

Our patient initially received three cycles of neoadjuvant chemotherapy using vincristine, actinomycin D, and cyclophosphamide (VAC), and later underwent a radical cystectomy associated with extended pelvic lymph node dissection with transileal urinary diversion (Bricker type). No lymph node metastasis was found and the margins of the resection were also negative. Our patient was free of disease 24 months after treatment.

## Discussion

RMS is a malignant tumor that arises from a normal skeletal muscle cell. RMS in the urinary bladder has been well documented in children and adolescents. The majority of these are embryonal RMS, predominantly the botryoid subtype [4, 5]. RMS of the urinary bladder in adults is distinctively rare. We report our experience of a rare case of RMS of the bladder in a 45-year-old man who was successfully managed with neoadjuvant chemotherapy and surgical excision of the mass. To the best of our knowledge, there are only a few adult cases of RMS of the urinary bladder that have been reported in the English literature [6–8], and our case is the first to report successful management with neoadjuvant chemotherapy and surgery like the management of urothelial bladder cancers [9].

In the reported cases, the tumor usually occurs in older patients with an average age of 63 ± 13 years. There is a male predominance with a male to female ratio of roughly 2 to 1 [10].

The tumor usually arises in the trigone and invades the surrounding tissue, presenting as a painful or painless mass. The tumor recurs frequently and metastasizes to the regional lymph nodes, lungs, or liver. The main symptoms of the disease are hematuria, dysuria, and, more generally, bladder dysfunction [8].

RMS in adults is composed of small round blue cells with a high nuclear cytoplasm ratio, brisk mitosis, and frequent apoptosis. Frequently, the tumors show nuclear anaplasia, with random large anaplastic cells scattered in the tumor, similar to the nuclear anaplasia seen in Wilms tumor without the requirement of tripolar atypical mitosis [11]. Sometimes, they show signs of striated muscle differentiation and become more elongated with a more abundant and eosinophilic cytoplasm. They are called “tadpoles” or “tennis-racket” cells [12]. RMS in adult urinary bladders has been reported to be embryonal [13], alveolar [13], pleomorphic [14], or unspecified type [15]. It should be noted that staining of the cells with anti-myogenin antibody is generally weaker and less diffuse in embryonic RMS than in the alveolar type. This helps the differential diagnosis with this last entity [12].

Embryonal RMS has three histopathologic variants that can occur singly or in combination: botryoid, spindled, and anaplastic. The botryoid variant, by definition, arises beneath epithelial mucosal surfaces and has a cambium layer (Fig. 2). The spindled variant occurs most commonly in scrotal soft tissues, with the remainder occurring in the head and neck region [16].

Positive immunohistochemical staining with anti-myoglobin antibody and anti-sarcomeric actin antibody, which is characteristic of striated muscles, is very helpful in the diagnosis of RMS [17].

For our patient, the pathological examination of the specimen showed embryonal RMS with botryoid subtype composed of intensely eosinophilic polygonal cells with strong diffuse desmin expression and strong diffuse immunoreactivity for vimentin and nuclear staining for myogenin (Figs. 4, 5, and 6).

Immunohistochemical studies showed specific nuclear transcription factors: Myo-D1, myogenin, and desmin. No cytokeratin or chromogranin reactivity was detected in these tumors.

Typically, the differential diagnosis of RMS in adults includes sarcomatoid urothelial carcinoma with extensive rhabdomyosarcomatous differentiation and other tumors with small round cell morphology including small cell carcinoma, plasmacytoid urothelial carcinoma, primitive neuroectodermal tumor, and lymphoma. Immunohistochemical analysis has an important role in the differential diagnosis of these tumors. Desmin and myogenin immunoreactivity can be used to differentiate RMS from other round cell tumors without rhabdomyoblastic differentiation but not sarcomatoid carcinoma with rhabdomyoblastic differentiation [10].

The prognosis and optimal management of adult embryonal RMS is uncertain due to its rarity [18]. For adult bladder RMS, treatments are variable, including surgical resection, radiotherapy, chemotherapy, or combined therapy.

The prognosis is poor in the majority of patients. A previous reported case on this disease highlighted that three patients who had undergone cystectomy and two other patients who had radiation therapy died 3 to 19 months after their treatments [19]. The worst prognostic factors for this disease include nonembryonal histology, tumor invasion, and tumor size > 5 cm in children with RMS [20].

As these tumors are more common in children and by referring to the management of these tumors in some centers [21], we found that chemotherapy was used as monotherapy in children with stage 2 embryonal RMS of the bladder/prostate with the advantage of bladder preservation. Adjuvant therapy or intensive chemotherapy might be required for stage 3 embryonal RMS [21]. By referring to the latest recommendation on the management of urothelial bladder cancers [9], our patient was treated with neoadjuvant chemotherapy using VAC. Our patient subsequently underwent a radical cystectomy associated with extended pelvic lymph node dissection. A histopathological examination of the specimen found no lymph node metastasis and the margins of the surgical specimen were negative for tumor cells. Our patient had no disease recurrence 24 months after surgery.

Given the good clinical and radiological outcomes in our case, neoadjuvant chemotherapy with a complete surgical excision should be currently considered one of the treatments of choice in this type of malignancy.

Further study using a large number of patients with a greater longitudinal follow up is required.

## Conclusions

To the best of our knowledge, our case is the first to report the successful management of a rare embryonal RMS of the urinary bladder with neoadjuvant chemotherapy and surgery. However, further studies using a large number of patients with a greater duration of follow up is required.
